# Achieving Reliable Peripheral Nerve Blockade in Cardiogenic Shock With Distorted Femoral Anatomy: A Case Report

**DOI:** 10.7759/cureus.116372

**Published:** 2026-09-16

**Authors:** Mohammed I Hakami, Ayman H Mustafa

**Affiliations:** 1 Anesthesiology, King Faisal Specialist Hospital and Research Centre, Riyadh, SAU

**Keywords:** aortic stenosis, cardiogenic shock, dilated cardiomyopathy, femoral hematoma, nerve stimulation, peripheral nerve block, regional anesthesia, ultrasound guidance

## Abstract

Patients with cardiogenic shock and severe structural heart disease may poorly tolerate both general and neuraxial anesthesia. In selected cases, peripheral nerve blockade may reduce hemodynamic disturbance, but the block must be reliable when pain, sympathetic stimulation, or emergency conversion to general anesthesia could be poorly tolerated.

We report a 62-year-old woman with cardiogenic shock, low-flow, low-gradient severe aortic stenosis, advanced dilated cardiomyopathy, pulmonary edema, orthopnea, and active femoral bleeding who required urgent femoral artery and vein repair. General anesthesia and neuraxial anesthesia were considered hazardous. Hematoma and anasarca distorted the femoral anatomy and prevented direct femoral nerve visualization. Combined ultrasound guidance and nerve stimulation were used to support safe needle advancement and functional nerve localization. Surgery proceeded with minimal sedation and without escalation of vasoactive support. This case highlights the role of reliability-focused peripheral nerve blockade when conventional anesthetic options are high risk and regional anatomy is distorted.

## Introduction

Urgent surgery in patients with cardiogenic shock and competing cardiac physiology presents a major anesthetic dilemma. Severe aortic stenosis requires preservation of preload, afterload, and coronary perfusion, whereas advanced dilated cardiomyopathy requires avoidance of myocardial depression and excessive afterload [1,2].

General anesthesia may worsen this balance through vasodilation, myocardial depression, airway stimulation, and positive pressure ventilation; neuraxial anesthesia may produce abrupt sympathectomy. Peripheral nerve blockade may minimize systemic hemodynamic disturbance, but its reliability becomes critical when failed analgesia or emergency conversion to general anesthesia may be poorly tolerated. This challenge is amplified when hematoma and edema distort regional anatomy and prevent direct nerve visualization.

Femoral access complications after cardiac catheterization are often self-limited, but expanding hematoma or active bleeding may require urgent vascular intervention [3].

We describe combined ultrasound and nerve stimulation to achieve femoral, ilioinguinal, and iliohypogastric blockade for urgent femoral vascular repair in a patient with cardiogenic shock, low-flow, low-gradient severe aortic stenosis, advanced dilated cardiomyopathy, active bleeding, and distorted femoral anatomy.

## Case presentation

A 62-year-old woman with rheumatic heart disease diagnosed at the age of 28 years, mechanical mitral valve replacement 21 years ago, tricuspid valve repair, valvular atrial fibrillation, prior stroke, chronic kidney disease, severe aortic stenosis with moderate aortic regurgitation, and heart failure with reduced ejection fraction was admitted with community-acquired pneumonia complicated by acute decompensated heart failure and acute kidney injury. Her home medications included lisinopril, bisoprolol, warfarin, spironolactone, and high-dose furosemide. Chest radiography demonstrated cardiomegaly with diffuse bilateral pulmonary edema (Figure 1).

**Figure 1:**
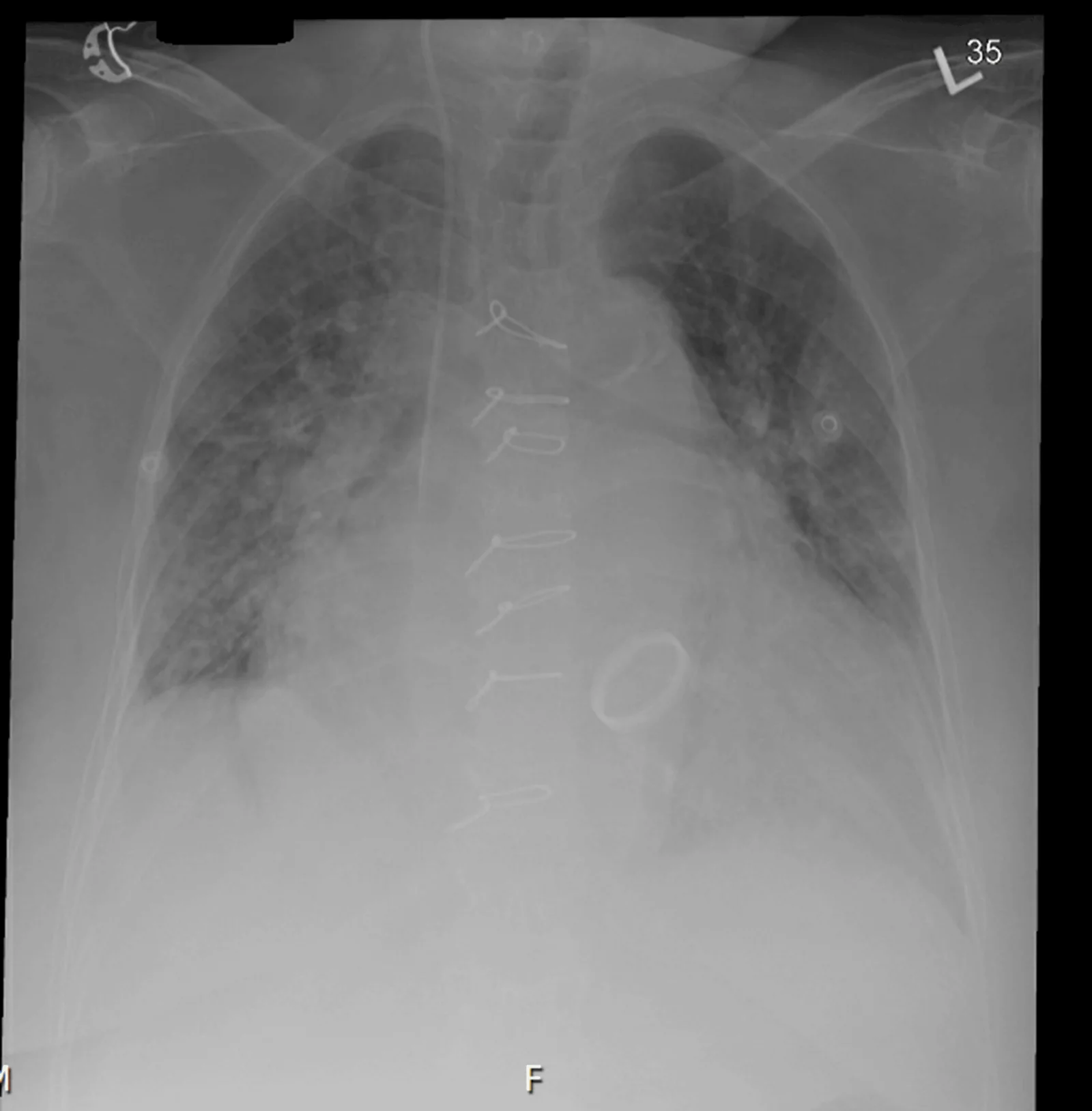
Portable anteroposterior chest radiograph Portable anteroposterior chest radiograph showing marked cardiomegaly with bilateral pulmonary vascular congestion and diffuse pulmonary edema. Sternotomy wires and a prosthetic mitral valve are visible.

Despite aggressive medical therapy, she deteriorated into cardiogenic shock requiring norepinephrine and milrinone. Transthoracic echocardiography, as reported by the cardiology team, demonstrated severe left ventricular dilation, global hypokinesia, left ventricular ejection fraction of 15%-20%, global longitudinal strain of -5.6%, moderate-to-severe tricuspid regurgitation, right ventricular systolic pressure of 50 mmHg, moderate right ventricular dilation with reduced systolic function, severe left atrial dilation, severe aortic stenosis with aortic valve area of 0.45 cm² and mean gradient of 34 mmHg, and moderate aortic regurgitation. Cardiac index was markedly reduced at 0.78 L/min/m², consistent with profound low-output physiology.

Diagnostic left heart catheterization was performed for hemodynamic assessment and evaluation of possible aortic valve intervention. Two days later, she developed progressive right groin pain and swelling, a decrease in hemoglobin requiring six units of packed red blood cells, and discontinuation of warfarin. Ultrasound and computed tomography confirmed a large right femoral hematoma with active bleeding (Figure 2). 

**Figure 2:**
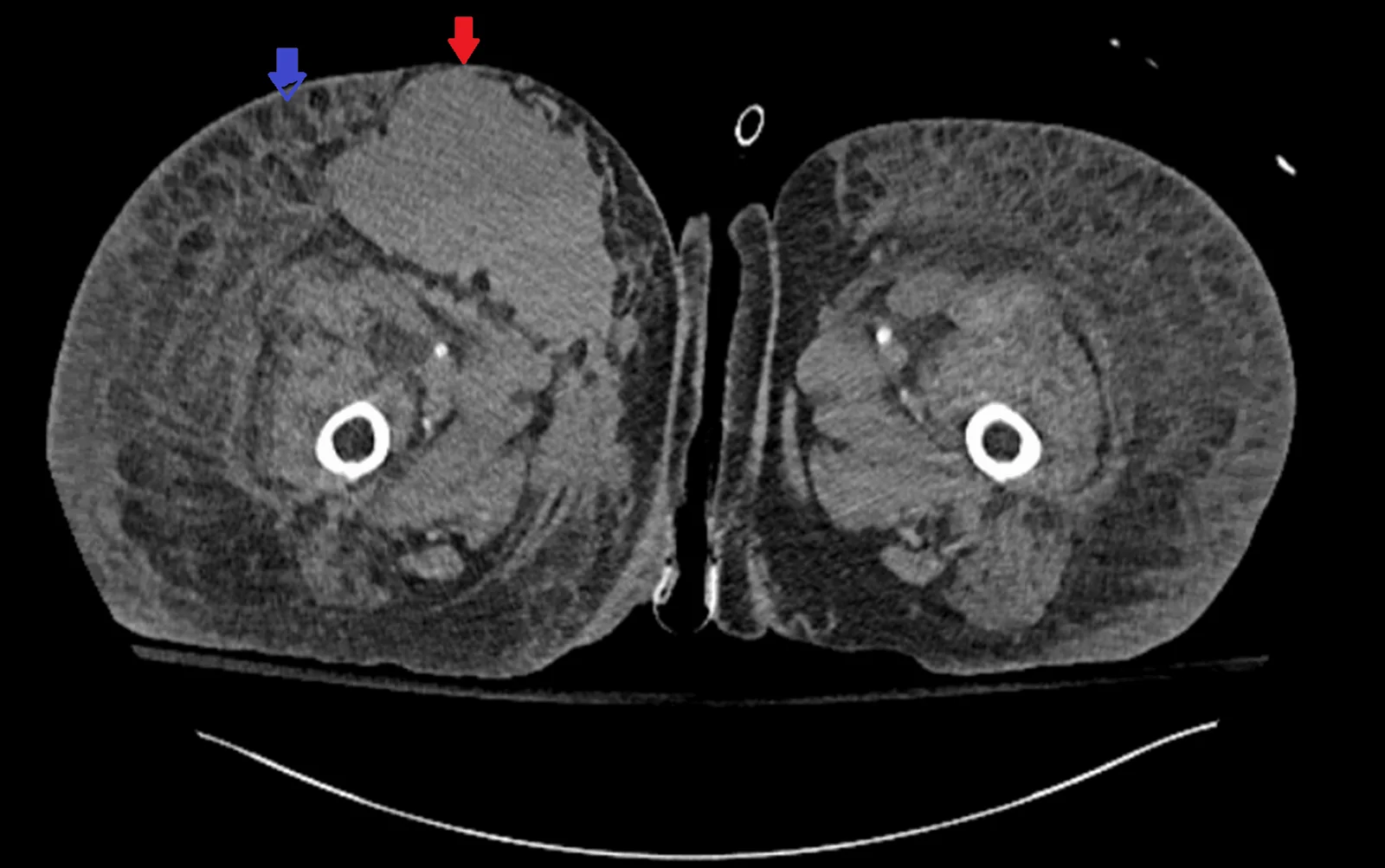
Axial computed tomography image of the proximal thigh and groin Axial computed tomography image of the proximal thigh and groin showing a large right femoral hematoma (red arrow) with diffuse soft-tissue edema (blue arrow) and distortion of regional anatomy.

Cardiac computed tomography also demonstrated a newly detected left atrial thrombus after interruption of anticoagulation despite not administering reversal to avoid such complications. Measured international normalized ratio (INR) dropped from 2.5 to 1.6 (reference range 0.9-1.1), and activated partial thromboplastin time (aPTT) dropped from 64 to 39 seconds (reference range 30.5-40.4 seconds). Endovascular management was not feasible, and urgent hematoma evacuation with femoral artery and vein repair was planned.

Preoperatively, she was in moderate respiratory distress with orthopnea requiring head elevation and supplemental oxygen by nasal cannula. She had generalized anasarca and severe bilateral lower-limb pitting edema. Invasive blood pressure measurement was 72/48 mmHg while receiving norepinephrine 0.1 µg/kg/min, milrinone 0.125 µg/kg/min, and furosemide infusion. Heart rate ranged between 90 and 115 beats/min, and respiratory rate ranged between 20 and 25 breaths/min.

The anesthetic goal was to avoid reductions in systemic vascular resistance, preload, and myocardial contractility while preserving spontaneous ventilation. Percutaneous valvular intervention or mechanical circulatory interventions were not pursued after multidisciplinary review because active bleeding, moderate aortic regurgitation, femoral vascular injury, and absence of an accepted definitive valve option made escalation unlikely to provide durable benefit.

General anesthesia was possible but hazardous. Induction could precipitate hypotension, airway stimulation could provoke tachycardia, positive pressure ventilation could reduce venous return and increase right ventricular afterload, and postoperative ventilatory difficulty was a possible postoperative concern in the presence of orthopnea and pulmonary edema. Neuraxial anesthesia was considered unsafe because sympathectomy could be poorly tolerated, and recent anticoagulation interruption with active bleeding and shock physiology further complicated the risk-benefit assessment.

Peripheral nerve blockade was therefore selected as the primary anesthetic strategy. However, a failed block was also dangerous. Pain-induced tachycardia, catecholamine release, and increased myocardial oxygen demand could precipitate cardiovascular collapse, while emergency conversion to general anesthesia after painful stimulation would likely be more hazardous than a controlled general anesthetic plan. Coupled with a challenging femoral anatomy, this further complicates decision-making in these situations.

Under invasive monitoring with the patient positioned with head elevation due to orthopnea, norepinephrine and milrinone infusions were continued, and one unit of packed red blood cells was transfused, targeting hemoglobin more than 10 g/dL (reference range, 120-160 g/dL). Femoral, ilioinguinal, and iliohypogastric nerve blocks were performed with a 100-mm Pajunk insulated nerve block needle under ultrasound guidance using a total of 30 mL ropivacaine 0.375%: 20 mL for the femoral nerve block and 10 mL for the ilioinguinal/iliohypogastric blocks, corresponding to a total ropivacaine dose of 112.5 mg, which corresponded to 1.3 mg/kg.

The femoral block was technically difficult. Severe anasarca and hematoma degraded ultrasound image quality; the femoral vein was noncompressible and pulsatile from venous congestion and severe tricuspid regurgitation, the artery and vein were separated by hematoma, and the femoral nerve was not visualized. The expected location of the femoral nerve immediately lateral to the femoral artery was therefore no longer reliable. The only feasible approach was to maintain continuous ultrasound visualization of the needle to avoid vascular injury while using adjunct nerve stimulation for functional localization of a probable femoral nerve location. Stimulation was initially set at approximately 1.0 mA, and the needle was advanced slowly under ultrasound visualization. After a motor response was obtained, the nerve stimulator current was gradually reduced while optimizing needle position. A satisfactory femoral nerve motor response was maintained at approximately 0.5 mA in a markedly lateral region of the groin, well lateral to the femoral artery and at an approximate depth of 4.5-5 cm from the skin, after which local anesthetic was injected.

The contingency plan for inadequate blockade included surgical local anesthetic supplementation, cautious incremental sedation, vasoactive escalation, and conversion to general anesthesia only as a last resort. Surgical incision began approximately 20 minutes after completion of the blocks, following confirmation of adequate anesthesia in the operative field using a sharp object.

Sedation was deliberately titrated in very small increments because both hypoventilation and sympathetic stimulation were poorly tolerated, and reduced cardiac output was expected to increase sensitivity to sedative and analgesic drugs. Surgery proceeded with fentanyl 25 µg administered gradually over 30 minutes and ketamine 2 mg. Intraoperatively, the patient remained hemodynamically stable; no clinically significant hemodynamic instability was documented, and no escalation of vasoactive support was required during the procedure. Estimated blood loss was 500 mL.

She was transferred to the cardiovascular intensive care unit hemodynamically stable and pain-free. Her code status was later changed to do-not-resuscitate after multidisciplinary discussion because of end-stage cardiac disease. Despite maximal medical therapy, her cardiac condition progressively deteriorated, and she died two weeks after surgery.

## Discussion

This case illustrates a high-stakes regional anesthesia dilemma rather than simply the use of peripheral nerve blockade in a high-risk cardiac patient. The patient’s severe cardiac disease created a narrow physiologic margin. Severe aortic stenosis requires maintenance of preload, systemic vascular resistance, and coronary perfusion, whereas advanced dilated cardiomyopathy requires preservation of contractility and avoidance of excessive afterload [1,2,4]. In low-flow, low-gradient aortic stenosis with reduced ejection fraction, the transvalvular gradient may underestimate anatomic severity because stroke volume is critically reduced [4,5]. This physiology leaves little tolerance for anesthetic-induced reductions in preload, afterload, or contractility.

General anesthesia remained possible, but it carried substantial risk. Induction may cause vasodilation and myocardial depression, laryngoscopy may provoke tachycardia and increased myocardial oxygen demand, and positive pressure ventilation may reduce venous return and increase right ventricular afterload [6]. These effects were particularly relevant because the patient had pulmonary edema, orthopnea, biventricular dysfunction, and cardiogenic shock. Neuraxial anesthesia avoided airway manipulation but introduced the risk of abrupt sympathectomy, which could be catastrophic in severe aortic stenosis and shock.

Peripheral nerve blockade was therefore selected as the least physiologically disruptive anesthetic option, but only a reliable block would be acceptable. Failed blockade would not merely cause discomfort; it could trigger sympathetic activation, tachycardia, increased myocardial oxygen demand, and cardiovascular collapse. Emergency induction of general anesthesia after failed blockade and painful stimulation would likely carry greater risk than planned general anesthesia. This changed the purpose of the regional technique: the block was not simply an analgesic intervention, but a hemodynamic strategy that needed to be dependable.

The technical problem was that the same pathology requiring surgery also made regional anesthesia difficult. Hematoma and anasarca distorted the femoral neurovascular anatomy and degraded ultrasound image quality. The vein was noncompressible and pulsatile, likely reflecting venous congestion and tricuspid regurgitation. The artery and vein were separated by hematoma, and the femoral nerve was not visualized. In this setting, combined ultrasound and nerve stimulation served complementary roles. Ultrasound was used primarily for needle and vascular safety, whereas nerve stimulation provided functional localization when direct sonographic nerve identification failed [7].

The hematoma also introduced uncertainty regarding local anesthetic risk. Direct evidence on local anesthetic pharmacokinetics during peripheral nerve blockade adjacent to hematoma is limited. However, rapid systemic lidocaine absorption has been reported after injection into fracture hematoma, and local anesthetic systemic toxicity risk is influenced by total dose, tissue vascularity, inadvertent intravascular injection, and patient-specific susceptibility [8-10]. In a patient with cardiogenic shock and severe structural heart disease, even modest systemic local anesthetic exposure could be poorly tolerated. These considerations supported dose restraint, avoidance of vascular puncture, fractionated injection, and close monitoring.

Sedation required the same reliability-focused approach. In cardiogenic shock, reduced cardiac output may alter both pharmacokinetics and pharmacodynamics by limiting early drug distribution, reducing hepatic and tissue perfusion, and increasing central or effect-site drug exposure after small boluses [11,12]. These effects are clinically relevant for sedative and analgesic agents whose distribution and clearance are influenced by perfusion. Fentanyl was therefore administered gradually, and ketamine was limited to a very small dose. Although ketamine may preserve spontaneous ventilation and often supports blood pressure through indirect sympathetic stimulation, it can also increase heart rate and myocardial oxygen demand, and its direct myocardial depressant effect may become more apparent in severe myocardial dysfunction or catecholamine-depleted states [13]. The patient’s adequate response to fentanyl 25 µg over 30 minutes and an unexpectedly low dose of ketamine (2 mg) supports titration to effect rather than use of routine procedural sedation doses in profoundly low-output states, specifically with concerns for exaggerated hemodynamic or ventilatory effects.

This case also highlights the importance of planning for failure. When regional anesthesia is chosen because general and neuraxial anesthesia are hazardous, the threshold for accepting an uncertain block should be low. Rescue options should be explicit before incision, including surgical local anesthetic supplementation, cautious incremental sedation, vasoactive escalation, and criteria for conversion to general anesthesia. In this patient, achieving an effective block before surgical stimulation was essential because both failed analgesia and emergency conversion carried major hemodynamic risk. This decision-making approach is consistent with contemporary multi-society perioperative cardiovascular guidance, which emphasizes individualized cardiovascular risk assessment, multidisciplinary planning, and risk mitigation in adults undergoing noncardiac surgery [14].

This report also has limitations. It describes a single patient and cannot establish the superiority or general safety of combined ultrasound and nerve stimulation compared with other anesthetic strategies. Block success was assessed clinically rather than by a standardized sensory-motor scoring system, and no ultrasound image of the final needle position was available. Local anesthetic plasma concentrations were not measured, and therefore conclusions regarding systemic absorption or LAST risk remain inferential. Finally, the patient’s subsequent death reflected progressive end-stage cardiac disease and does not allow conclusions about longer-term outcome after this anesthetic approach.

## Conclusions

In selected patients with cardiogenic shock and conflicting cardiac physiology, peripheral nerve blockade may serve as a hemodynamic strategy when general and neuraxial anesthesia are poorly tolerated. However, this approach is only useful if the block is reliable. When hematoma and edema distort anatomy and prevent nerve visualization, combined ultrasound and nerve stimulation may provide complementary technical support: ultrasound assists needle visualization and vascular avoidance, while nerve stimulation provides functional localization. This case emphasizes that in unstable patients, regional anesthesia planning must include block reliability, local anesthetic toxicity risk, altered sedative pharmacology, and a predefined rescue plan.
